# Structural and haemodynamic evaluation of less invasive surfactant administration during nasal intermittent positive pressure ventilation in surfactant-deficient newborn piglets

**DOI:** 10.1371/journal.pone.0284750

**Published:** 2023-04-28

**Authors:** Victoria Mielgo, Elena Gastiasoro, Fabrizio Salomone, Francesca Ricci, Miguel A. Gomez-Solaetxe, Lara Olazar, Begoña Loureiro, Carmen Rey-Santano

**Affiliations:** 1 Animal Research Unit, Biocruces-Bizkaia Health Research Institute, Barakaldo, Bizkaia, Spain; 2 Primary Health Care, Biocruces-Bizkaia Health Research Institute, Barakaldo, Bizkaia, Spain; 3 Chiesi Farmaceutici, R&D Department, Parma, Italy; 4 Medical Devices Group, University of the Basque Country (EHU), Portugalete, Bizkaia, Spain; Sohag University Faculty of Medicine, EGYPT

## Abstract

The most recent approaches to the initial treatment of respiratory distress syndrome (RDS)- involve non-invasive ventilation (NIV) and less-invasive surfactant (SF) administration (LISA). Combining these techniques has been proven a useful treatment option for SF-deficient neonates. The objective of this study was to explore the impact on the brain (using cerebral near infrared spectroscopy, NIRS) of different LISA methods during NIV, using nasal intermittent positive pressure ventilation (NIPPV) for treating neonatal RDS. For this, we used five groups of spontaneously breathing newborn piglets (n = 6/group) with bronchoalveolar lavage (BAL)-induced respiratory distress which received NIPPV only (controls), poractant-alfa using the INSURE-like method (bolus delivery) followed by NIPPV, or poractant-alfa using one of three LISA devices, 1) a nasogastric tube (NT), 2) a vascular catheter (VC) or 3) the LISAcath® catheter. We assessed pulmonary, hemodynamic and cerebral effects, and performed histological analysis of lung and brain tissue. Following BALs, the piglets developed severe RDS (pH<7.2, P_aCO2_>70 mmHg, P_aO2_<70 mmHg, dynamic compliance<0.5 ml/cmH_2_O/kg at F_iO2_ = 1). Poractant-alfa administration using different LISA techniques during NIPPV was well tolerated and efficacious in newborn piglets. In our study, although all groups showed normal physiological ranges of total lung injury score and biochemical lung analysis, VC and LISAcath® catheters were associated with better values of lung compliance and lower values of lung damage than NIPPV, NT or INSURE-like methods. Moreover, neither of the SF administration methods used (LISA or INSURE-like) had a significant impact on the histological neonatal brain injury score. Of note, the LISAcath® has been recently withdrawn from the market.

## Introduction

The search for alternatives to classical bolus surfactant (SF) therapy administered through an endotracheal tube during invasive mechanical ventilation for the treatment of neonatal respiratory distress syndrome (RDS) has led to the development of INtubation–SURfactant–Extubation (INSURE) and less invasive SF administration (LISA) methods. Currently, the most widely used LISA method involves SF administration via a thin catheter, either employing a nasogastric tube (NT), a vascular catheter (VC) or a LISAcath® catheter in spontaneously breathing neonates during non-invasive ventilation (NIV). Until now, most preclinical and clinical studies evaluating the feasibility and benefits of the LISA method using thin catheters have been conducted during nasal continuous positive airway pressure (NCPAP) [1–4]. Nonetheless, the most recently published randomized trial in premature neonates with RDS was planned to compare the efficacy of SF administration using a NT and the INSURE method while on nasal intermittent positive pressure ventilation (NIPPV) as the primary mode of NIV instead of NCPAP [5], confirming that NIPPV is feasible and might be helpful during LISA [5–7].

It is well known that during SF administration transient episodes of desaturation and bradycardia may occur, and hence, it is crucial to protect the immature brain from prolonged episodes of hypoxemia to avoid adverse neurodevelopmental outcomes [8]. Studies have demonstrated the potential benefit of using near-infrared spectroscopy (NIRS) for assessing regional cerebral tissue oxygenation saturation/index (rcSO_2_ or cTOI) as a biomarker of brain vulnerability [9, 10], but only one [11] has evaluated the effect of the LISA method during NCPAP on cerebral oxygenation. Further, no one has explored potential differences in the effect on the neonatal brain of using the INSURE-like method, or a LISA method with NT, VC or LISAcath® catheters for SF administration during NIPPV, as the primary respiratory support.

Our hypothesis was that combination of NIPPV and SF administration, using any of the tested catheters (NT, VC and LISAcath®) for LISA, would result in a good physiological response similar to or better than that observed after SF administration by the INSURE-like method. Our main objective therefore was to compare these aforementioned methods of administering SF, including evaluation of their short-term impact on the brain in development using NIRS, in spontaneously breathing newborn piglets with respiratory distress induced by bronchoalveolar lavage (BAL). In addition, we explored responses to the combination of these two non-invasive treatments (use of LISA plus NIPPV) as reflected in gas exchange and hemodynamics, oxygen metabolism and lung injury scores.

## Material and methods

### Animal preparation

The study was approved by the Ethics Committee for Animal Welfare of Biocruces-Bizkaia Health Research Institute (OEBA-CET-2019-001) and all experiments adhered to both Spanish and European regulations for research in animals (UE2010/63-RD53/2013) and followed the ARRIVE guidelines 2.0.

In brief, 2- to 4-day-old newborn piglets [12, 13] (Topig 20, Large-White and Landrace Hybrid F1, Arri-Turri Farm, Alava, Spain) were sedated with ketamine (15 mg/kg), diazepam (2 mg/kg) and atropine (0.05 mg/kg) i.m. and anesthetized with sevoflurane (2–3%). They were then put on ventilation with a positive pressure ventilator (VIP Bird, Bird Products Corp., Palm Springs, CA) through a cuffed endotracheal tube and initial settings of F_iO2_ of 0.21–0.28, respiratory rate (RR) of 28 breaths/min, positive end-expiratory pressure (PEEP) of 3 cmH_2_O and positive inspiratory pressure (PIP) of 9–11 cmH_2_O adjusted to achieve a tidal volume (VT) of 8–10 mL/kg [14–16].

Two catheters were placed, one into the femoral artery to measure mean arterial blood pressure (MABP) and heart rate (HR) and collect blood samples for gas analysis, and one into the external jugular vein, to give fluid therapy and take venous blood samples. Further, an ultrasonic flow probe (Transonic Systems Inc., NY) was employed to assess common right carotid blood flow as a proxy for cerebral blood flow [17]. Lastly, a sensor was fixed to the frontoparietal region of each piglet´s head for the NIRS using NIRO-200 system (Hamamatsu Photonics, Joko Cho, Japan), to detect changes in cerebral perfusion and oxygenation. Heat lamps were used to keep the rectal temperature at 38–39°C.

### Study design including induction of lung injury

**Fig 1:**
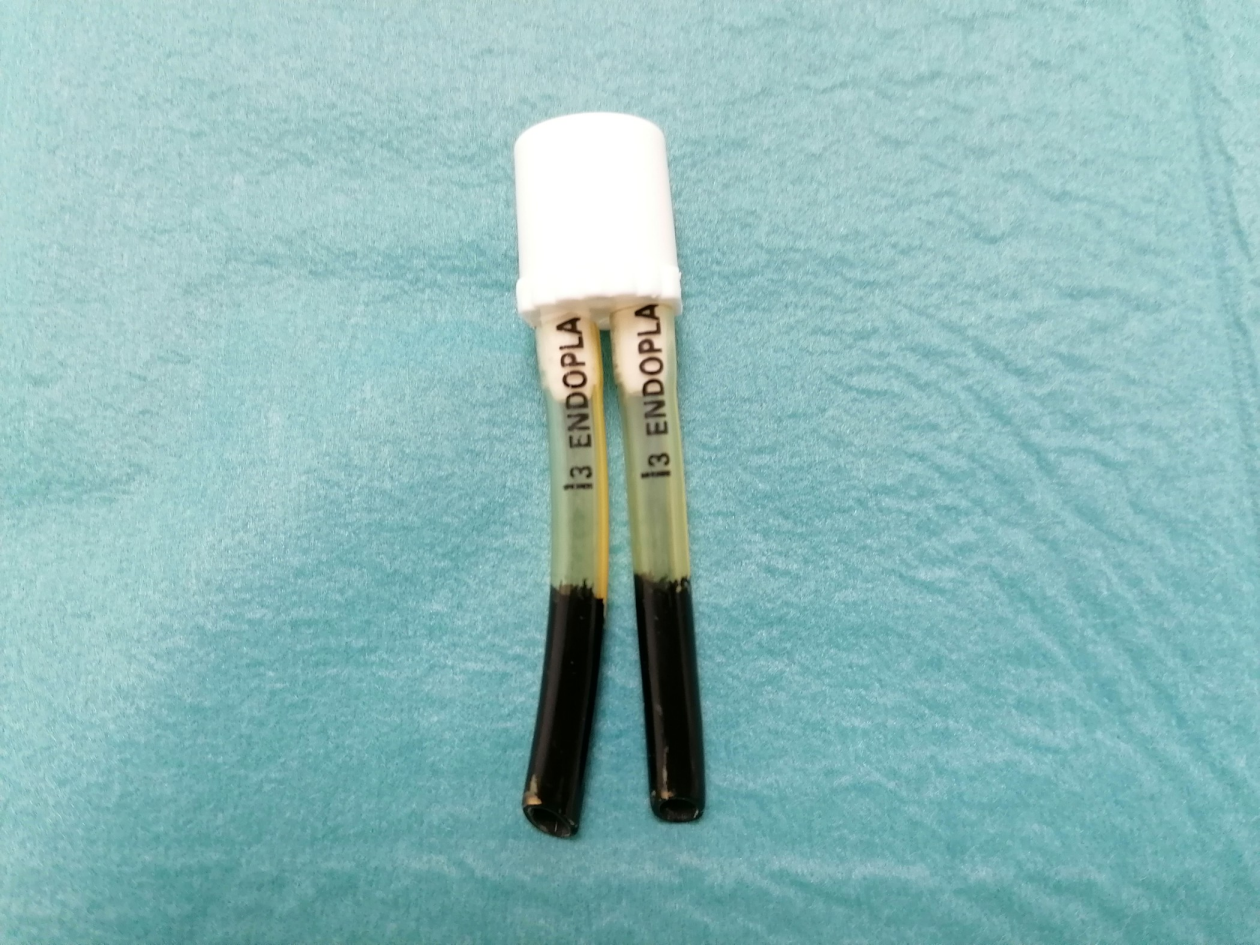
Short customized tightly-fitting binasal prongs.

A total of 30 newborn piglets were used in this study. The induction of SF-deficient lung injury was achieved by repeated BALs (30 ml/kg of saline every lavage; 37°C with F_iO2_:1) [15, 18]. During the lavage procedure, the positive pressure ventilator was set at: F_iO2_ = 1.0 [15, 18, 19] and PEEP = 5 cmH_2_O, and seeking to minimize barotrauma, RR was ajusted to ≤42 breaths/min and PIP ≤25 cmH_2_O, to keep VT within 8–10 ml/kg. Every 5 min, further lavage was performed until P_aO2_ had fallen to <100 mmHg F_iO2_ = 1.0 [15, 18, 19]. Piglets were allowed to stabilize for 30 min on positive pressure ventilation, and then given a 20 mg/kg bolus dose of caffeine citrate (Peyona® 20 mg/ml; Chiesi Farmaceutici, Parma, Italy) intravenously to stimulate spontaneous breathing and fitted with short customized tightly-fitting binasal prongs (made by cutting and joining two pieces of endotracheal tube, with an internal diameter of 4 mm and length of 4 cm, matched to the size of our piglets’ nasal orifice) (Fig 1).

Once breathing spontaneously, piglets were randomly assigned using a sealed envelope system to one of five groups:

- NIPPV group (n = 6): no SF was administered; the ET was removed and animals were maintained on NIPPV.
- INSURE-like (INSURE) group (n = 6): 200 mg/kg of poractant alfa (Curosurf®, Chiesi Farmaceutici, Parma, Italy) was administered in one minute through the ET, which was then immediately removed and the animals were switched to NIPPV.
- LISA-nasogastric tube (NT) group (n = 6): the ET was removed and 200 mg/kg of SF was administered in one minute by the LISA method using 5Fr flexible NT that had been appropriately shortened and placed in the trachea with direct visualization of the vocal cords with a laryngoscope and using Magill forceps.
- LISA-vascular catheter (VC) group (n = 6): the ET was removed and 200 mg/kg of SF was administered in one minute by the LISA method using a 5Fr vascular catheter (Seldicath®, Prodimed, Le Plessis-Bouchard, 5Fr) placed in the trachea with direct visualization of the vocal cords with a laryngoscope (without the use of Magill forceps).
- LISAcath® catheter (LISAcath) group (n = 6): the ET was removed and 200 mg/kg of SF was administered in one minute by the LISA method using a LISAcath® catheter (Chiesi Farmaceutici, Parma, Italy, 5Fr) placed in the trachea with direct visualization of the vocal cords with a laryngoscope (without the use of Magill forceps). Of note, the LISAcath® has been recently withdrawn from the market while elaborating this manuscript.

In all SF-treated groups, the SF used was poractant alfa (Curosurf®, Chiesi Farmaceutici, Parma, Italy) at a dose of 200 mg/kg. All groups were kept on NIPPV for 180 min following endotracheal tube removal. The NIPPV settings were initially: RR of 40 breaths/min; PEEP 5 cmH_2_O and PIP 15–17 cmH_2_O at F_iO2_ = 1. Subsequently, piglets were monitored and ventilator settings were adjusted on a case-by-case basis, to keep P_aO2_ between 80 and 100 mmHg and P_aCO2_ between 35 and 45 mmHg, respectively. As soon as P_aO2_ improved, and seeking to keep values in the aforementioned range, F_iO2_ was reduced, to minimize oxygen-induced lung injury. The same experienced researcher performed all LISA procedures to avoid introducing bias to the study.

### Lung and cardiovascular outcomes

#### Gas exchange and cardiovascular parameters

The following were assessed: arterial pH, P_aCO2_, P_aO2_/F_iO2_ ratio, oxygen delivery (OD), oxygen consumption (VO_2_), intrapulmonary-shunt (Qs/Qt) [14] and hemodynamic parameters, namely, HR and MABP (Intellivue MP70, Philips-Medical, Eindhoven, The Netherlands) were measured or calculated. Oxygen delivery (OD), oxygen consumption (VO_2_) and intrapulmonary shunt ratio (Qs/Qt) (IntelliVue Monitor; Philips Medical System), were calculated using the following equations:

- C_a(v)O2_ = (S_a(v)O2_ × Hb × 1.39/100) + (P_a(v)O2_ × 0.003); C_aO2_ is arterial O_2_ content and C_vO2_ is mixed venous O_2_ content.
- OD = C_aO2_ × cardiac output; and
- VO_2_ = (C_aO2_ − C_vO2_) × cardiac output.
- Qs/Qt (%) = 100 × (1.34 ×Hb + 0.0031 × P_AO2_ −C_aO2_)/(1.34×Hb+0.0031×P_AO2_ −C_vO2_), where Hb is hemoglobin (g/dl); P_AO2_ = F_iO2_ × (Patmospheric − 47) − P_aCO2_.

These parameters were measured or calculated at baseline, following the last BAL, during the period of stabilization under conventional mechanical ventilation (lasting 30 minutes), and then after extubation every 30 min during the 180-min on NIPPV.

#### Lung mechanics

These were assessed with a computerized system (M1014A, Philips Medical, Eindhoven, The Netherlands) that provides values for dynamic compliance (Cdyn). For these values, readings were recorded at baseline, following the last BAL, after 30 min of stabilization, and at the end of the experiment (for this purpose, the piglets were reintubated at 180 min after extubation, it not being possible to measure lung mechanics during NIPPV).

#### Lung tissue analysis

Postmortem, the left lung was prepared for biochemical analysis (isolated, occluded, submerged in liquid nitrogen, and stored at -80°C until use), and the right lung for histological analysis (fixed in 4% formalin at 15 cmH_2_O and subsequently, cut into 5-μm sections, placed on slides and hematoxylin-eosin stained). Specific enzyme-linked immunosorbent assay kits for porcine interleukins (Abnova, Tapei City, Taiwan) were used to assess IL-8, IL-1B, and TNF-α levels and the Bradford method (Bio-Rad, Hercules, CA) [20] to measure protein levels in samples taken from the frozen lung tissue. A pathologist blinded to group allocation assessed the formalin-fixed sections with light microscopy and rated the extent of each type of lung injury considered (atelectasis, alveolar and interstitial inflammation, alveolar and interstitial hemorrhage, edema, and necrosis) on a 0- to 4-point scale (0: no injury; 1, 2, and 3: injury to 25%, 50%, and 75% of the field, respectively; and 4: injury across the field; the total score ranging from 0 to 28) [15, 21], values higher than 12 corresponding to quite a severe lung injury.

### Cerebral outcomes

#### Carotid blood flow and NIRS measurements

Similar to other parameters, these measurements were taken at baseline, following the last BAL, after the 30 min of stabilization, and after extubation, every 30 min during the 180-min on NIPPV. Further, to explore potential changes during SF administration, the cerebral outcomes were also assessed at 2 min before (-2 min), during (0 min), and at 1 and 5 min after administering the SF. As mentioned earlier, carotid blood flow was measured as a proxy for cerebral perfusion and NIRS was used to monitor changes in cerebral perfusion and oxygenation. Specifically, we set up continuous monitoring of the cTOI, which represents the cerebral oxygen saturation expressed as a percentage. Further, this index was used to calculate the cerebral fractional tissue oxygen extraction (cFTOE) as follows: cFTOE = (S_pO2_-cTOI)/S_pO2_ [1].

#### Brain tissue analysis

For the histological analysis, the brain tissue was fixed in 4% formalin and cut into sections corresponding to the cortex, inner regions (striatum, thalamus, and hippocampus), and cerebellum and brain stem. The formalin-fixed tissue was cut into 5-μm sections, placed on slides and hematoxylin-eosin stained. As for the lung tissue, a pathologist blinded to group allocation assessed the sections with light microscopy, analyzing 20 fields, and in this case, rated pathological features of brain injury (necrosis, inflammation, hemorrhage, edema and infarction) on a 0- to 3-point scale (0: no injury; and 1, 2, and 3: mild, moderate, and severe injury across the field respectively; with a total score ranging from 0 to 15). Neuronal necrosis was defined as more than five necrotic cells/field [15].

### Statistical analysis

Results are expressed as mean±standard error of the mean (SEM). Levene’s test was used to check the homogeneity of variance between the groups and Kolmogorov-Smirnov test to assess whether the data were normally distributed (JMP8, Statistical Discovery, SAS, NC). One-way analysis of variance (ANOVA) was performed to assess differences in gas exchange, hemodynamic parameters, and brain evaluation parameters as a function of group. Comparisons of results (gas exchange, hemodynamic parameters and cerebral oxygen metabolism) were performed by repeated measures two-way ANOVA as a function of group and time. Short-term cerebral effects, and lung and brain injury scores were analyzed using the Wilcoxon nonparametric test. A p<0.05 was considered significant.

## Results

For this study, we used 30 newborn piglets which were similar in age (3±1days) and size (2.0±0.1kg), being alive at the end of the experimental period. Further, the numbers of BALs required (NIPPV group: 13±4; INSURE group: 13±3; NT group: 14±3; VC group: 15±2; LISAcath group: 16±2) to induce appropriately severe lung injury (P_aO2_<100 mmHg) (NIPPV group: 44±4 mmHg; INSURE group: 46±1 mmHg; NT group: 47±3 mmHg; VC group: 47±2 mmHg; LISAcath group: 46±3 mmHg, at F_iO2_ = 1) and the volume of lavage fluid recovered did not vary significantly between groups (S1 File).

### Pulmonary outcomes

#### Gas exchange and lung mechanics

**Fig 2:**
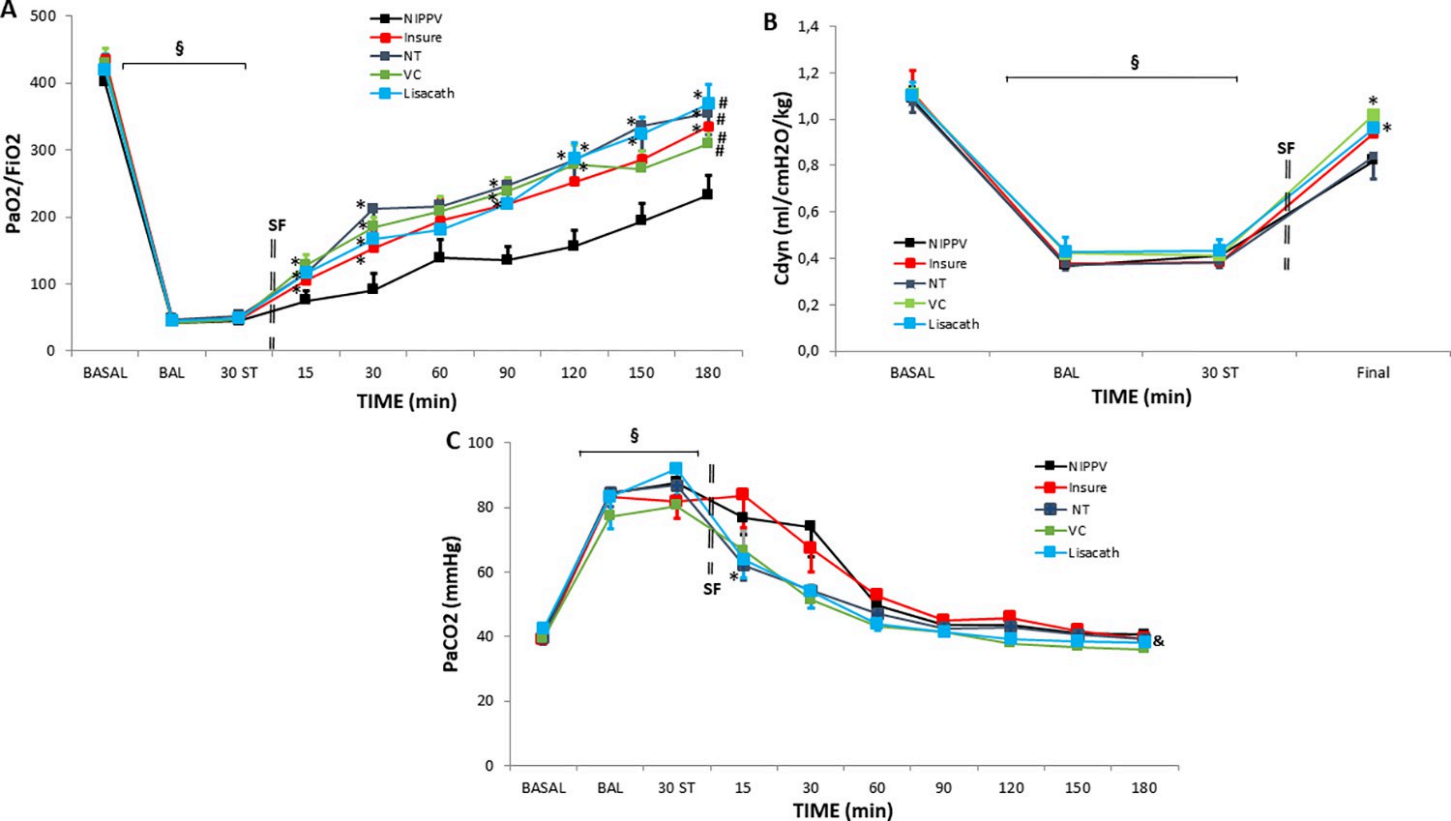
P_aO2_/F_iO2_ ratio, dynamic compliance (Cdyn) and P_aCO2_ in newborn piglets with broncoalveolar lavage-induced respiratory distress treated with nasal-intermittent-positive-pressure-ventilation (NIPPV) without SF therapy or with SF therapy, using the INSURE-like method, LISA with a nasogastric tube (NT), LISA with a vascular catheter (VC) or LISA with the LISAcath® catheter over the 180-min treatment period. Values of P_aO2_/F_iO2_ (A), Cdyn (B), and P_aCO2_ (C) in the NIPPV alone (black), INSURE (red), NT (dark-blue), VC (green) and LISAcath (blue) groups. The Cdyn value at 180 min was measured after the reintubation of the animals at the end of the study. (§)p<0.05 vs baseline; (*)p<0.05 vs NIPPV group (one-way-ANOVA); (#)p<0.05 vs NIPPV group and (&)p<0.05 vs INSURE group (two-way-ANOVA). Values = mean±SEM. ST: stabilization.

In measurements at baseline, after induction of lung injury and after stabilization for 30 minutes, pH, P_aO2_/F_iO2_, P_aCO2_ and Cdyn did not differ significantly between groups (Table 1 and Fig 2; S2 and S3 Files). Further, significant reductions in P_aO2_/F_iO2_ (Fig 2A), Cdyn (Fig 2B) and pH (Table 1) and a significant increase in P_aCO2_ (Fig 2C), consistent with severe respiratory distress, were observed in all groups after the lavages.

**Table 1:** pH, respiratory rate and oxygen metabolism in bronchoalveolar lavage-induced respiratory distress newborn piglets treated with nasal intermittent positive pressure ventilation (NIPPV) without SF therapy or with SF therapy, using the INSURE-like method, less-invasive SF administration (LISA) with a nasogastric tube (NT), LISA with a vascular catheter (VC) or LISA with the LISAcath® catheter over the 180-min treatment period.

	Groups	Baseline	BAL	30 ST	15 min	30 min	60 min	90 min	120 min	150 min	180 min	
**pH**	**NIPPV**	7.41±0.02	7.10±0.04§	7.05±0.06§	7.12±0.04	7.20±0.05	7.34±0.01	7.40±0.01	7.41±0.01	7.44±0.01	7.46±0.01	
	**INSURE**	7.38±0.02	7.10±0.01§	7.10±0.03§	7.12±0.04	7.20±0.03	7.31±0.01	7.37±0.03	7.38±0.03	7.41±0.02	7.43±0.02	
	**NT**	7.37±0.01	7.09±0.03§	7.07±0.03§	7.20±0.03	7.27±0.04	7.31±0.02	7.37±0.01	7.38±0.01	7.39±0.01*	7.42±0.01	
	**VC**	7.38±0.02	7.14±0.02§	7.08±0.02§	7.18±0.01	7.26±0.01	7.35±0.02	7.41±0.01	7.42±0.01	7.43±0.02%	7.44±0.02	
	**LISAcath**	7.37±0.01	7.10±0.03§	7.09±0.03§	7.21±0.03	7.27±0.04	7.34±0.02	7.41±0.02	7.42±0.02	7.45±0.02	7.44±0.02	
**Respiratory rate** (bpm)	**NIPPV**	28±1	42±0§	42±0§	58±10	58±10	65±8	58±7	50±5	47±5	50±4	
	**INSURE**	28±1	42±0§	42±0§	55±3	61±4	59±4	55±3	56±3	51±4	51±4	
	**NT**	28±1	42±0§	42±0§	74±3$	70±2$	58±7	49±3	43±5$	37±5$	40±6	#&
	**VC**	28±1	42±0§	42±0§	56±8%	50±6%	51±4	49±7	44±4$	49±5	41±3	
	**LISAcath**	28±1	42±0§	42±0§	71±4	71±4$	65±4	50±4	51±2	47±5	43±3	&+
**OD** (ml/min)	**NIPPV**	50±5	42±10	38±7	59±8	65±8	64±8	60±6	61±6	66±6	62±6	
	**INSURE**	55±7	43±5	42±5	67±9	70±10	72±7	79±7	69±6	71±6	71±5	
	**NT**	48±4	40±6	42±6	66±7	71±8	67±8	67±9	64±10	59±10	55±6	
	**VC**	54±5	49±6	50±11	72±15	78±13	78±12	79±13	77±13	75±10	72±8	
	**LISAcath**	50±8	40±6	40±7	62±8	71±7	73±10	74±10	73±8	77±10	70±8	
**VO**_**2**_ (ml/min)	**NIPPV**	10±1	14±4	12±4	15±3	13±2	12±2	10±1	11±1	12±2	12±2	
	**INSURE**	13±2	14±3	15±4	15±3	16±3	20±3*	17±3	17±2*	18±4	19±3	
	**NT**	10±1	10±1	9±1	13±1	14±1	14±1	15±1*	13±1	13±2	16±1	
	**VC**	11±3	12±4	14±6	19±5	21±4	19±4	19±4	17±4	22±3*	17±2	
	**LISAcath**	10±1	9±2	9±2	10±1	13±2	12±2	13±2	19±5	13±2	14±2	

Statistical differences (§) p<0.05 vs baseline point; (*) p<0.05 vs NIPPV group, ($) p<0.05 vs INSURE, (%) p<0.05 vs NT (one-way analysis of variance); (#) p<0.05 vs NIPPV group, (&) p<0.05 vs INSURE group, (+) p<0.05 vs VC group (two-way analysis of variance). Values are expressed as mean±SEM. NIPPV: nasal intermittent positive pressure ventilation; NT: nasogastric tube; VC: vascular catheter; OD: oxygen delivery; VO_2_: oxygen consumption.

SF administration using INSURE-like, NT, VC or LISAcath techniques ameliorated the symptoms of RDS, P_aO2_/F_iO2_, pH and P_aCO2_ values in all SF-treated groups following a similar pattern and indicating improvements over the course of the experiment. In contrast, in the NIPPV group, the parameters measured continued to be indicative of mild-to-moderate RDS (notably, the P_aO2_/F_iO2_ ratio remaining < 250 mmHg).

Notably, Cdyn returned to or close to baseline (85–90%) in INSURE, VC and LISAcath groups, but in one of the SF-treated groups, namely, the NT group, Cdyn recovery was weaker and similar to that in the NIPPV alone group (Cdyn only reaching 78% of baseline).

In order to avoid hypercapnia, for the first 15–30 min after SF administration, RR was higher in all LISA SF-treated groups (Table 1). After that, no significant between-group differences were detected.

#### Lung inflammatory markers and lung injury

In measurements taken 3 hours after initiating NIPPV, inflammatory cytokine levels were generally similar in all SF-treated groups to those seen in the group only given NIPPV (Table 2 and S4 File).

**Table 2:** Biochemical lung analysis.

Groups	IL-8 (pg/mg prot)	TNF-alpha (pg/mg prot)	IL-1Beta (pg/mg prot)
**NIPPV**	42±11	69±11	280±77
**INSURE**	27±2	90±8	202±9
**NT**	37±13	69±16	320±73
**VC**	24±6	67±20	288±65
**LISAcath**	30±6	48±12$	248±56

Results of biochemical lung analysis in newborn piglets with bronchoalveolar lavage-induced respiratory distress treated with nasal intermittent positive pressure ventilation (NIPPV) without SF therapy or with SF therapy, using the INSURE-like method, less-invasive SF administration (LISA) with a nasogastric tube (NT), LISA with a vascular catheter (VC) or LISA with the LISAcath® catheter after the 180-min treatment period. Values are expressed as mean±SEM. ($) p<0.05 vs. INSURE (one-way analysis of variance). IL: interleukin; TNF: tumor necrosis factor.

**Fig 3:**
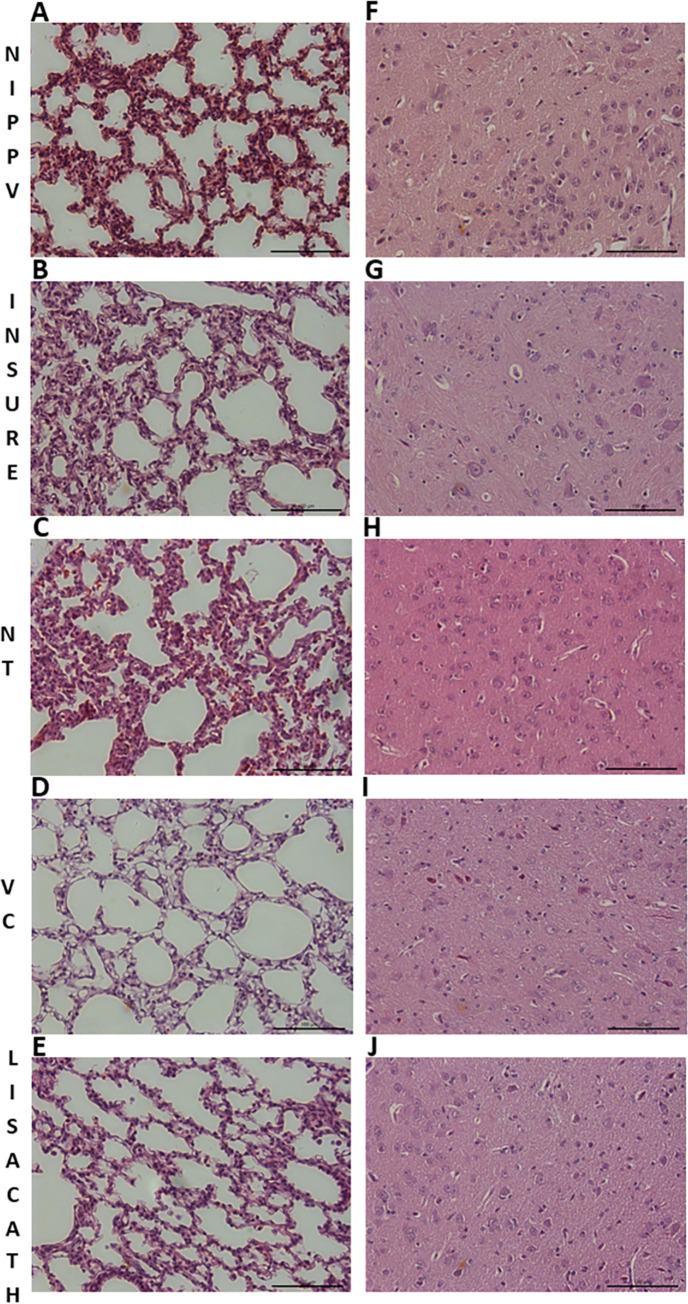
Photomicrographs (200x) of representative sections of the lung (A, B, C, D and E) and brain (F, G, H, I and J) from piglets in the NIPPV alone, INSURE, LISA with a nasogastric tube (NT), LISA with a vascular catheter (VC) and LISA with the LISAcath® catheter groups respectively. Lung sections were cut from the middle lobe of the lung and brain sections from the striatum. Bar represents 100 μm.

Although all groups showed normal physiological ranges of total lung injury score (Table 3 and S5 File) and biochemical lung analysis (Table 2 and S4 File), LISAcath group had significantly lower levels of TNF-alpha, less atelectasis, less edema, and lower total lung injury scores compared to levels in the INSURE and/or NIPPV groups (Fig 3).

**Table 3:** Total lung injury score.

Groups	Atelectasis	Necrosis	Edema	Alveolar inflammation	Interstitial inflammation	Alveolar hemorrhage	Interstitial hemorrhage	TOTAL
**NIPPV**	0.72±0.19	0.06±0.06	0.33±0.14	1.5±0.2	1.3±0.2	0.6±0.3	0.1±0.1	4.0±0.5
**INSURE**	0.61±0.22	0.06±0.06	0.08±0.06	1.0±0.2	1.3±0.2	0	0	3.0±0.5
**NT**	0.50±0.22	0	0.01±0.08	1.6±0.2	1.4±0.3	0	0	3.5±0.7
**VC**	0.47±0.17	0	0.07±0.07	1.1±0.2	1.8±0.2	0.3±0.2	0	2.7±0.6
**LISAcath**	0.11±0.08*^$^	0	0*	1.0±0.2	0.9±0.2	0.1±0.3	0	2.2±0.5*

Total lung injury scores measured in newborn piglets with bronchoalveolar lavage-induced respiratory distress treated with nasal intermittent positive pressure ventilation (NIPPV) without SF therapy or with SF therapy, using the INSURE-like method, LISA with a nasogastric tube (NT), LISA with a vascular catheter (VC) or LISA with the LISAcath® catheter after the 180-min treatment period. Values are expressed as mean±SEM. (*) p<0.05 vs. NIPPV. ($) p<0.05 vs. INSURE (one-way analysis of variance).

### Intrapulmonary shunt, hemodynamic assessment and oxygen transport

**Fig 4:**
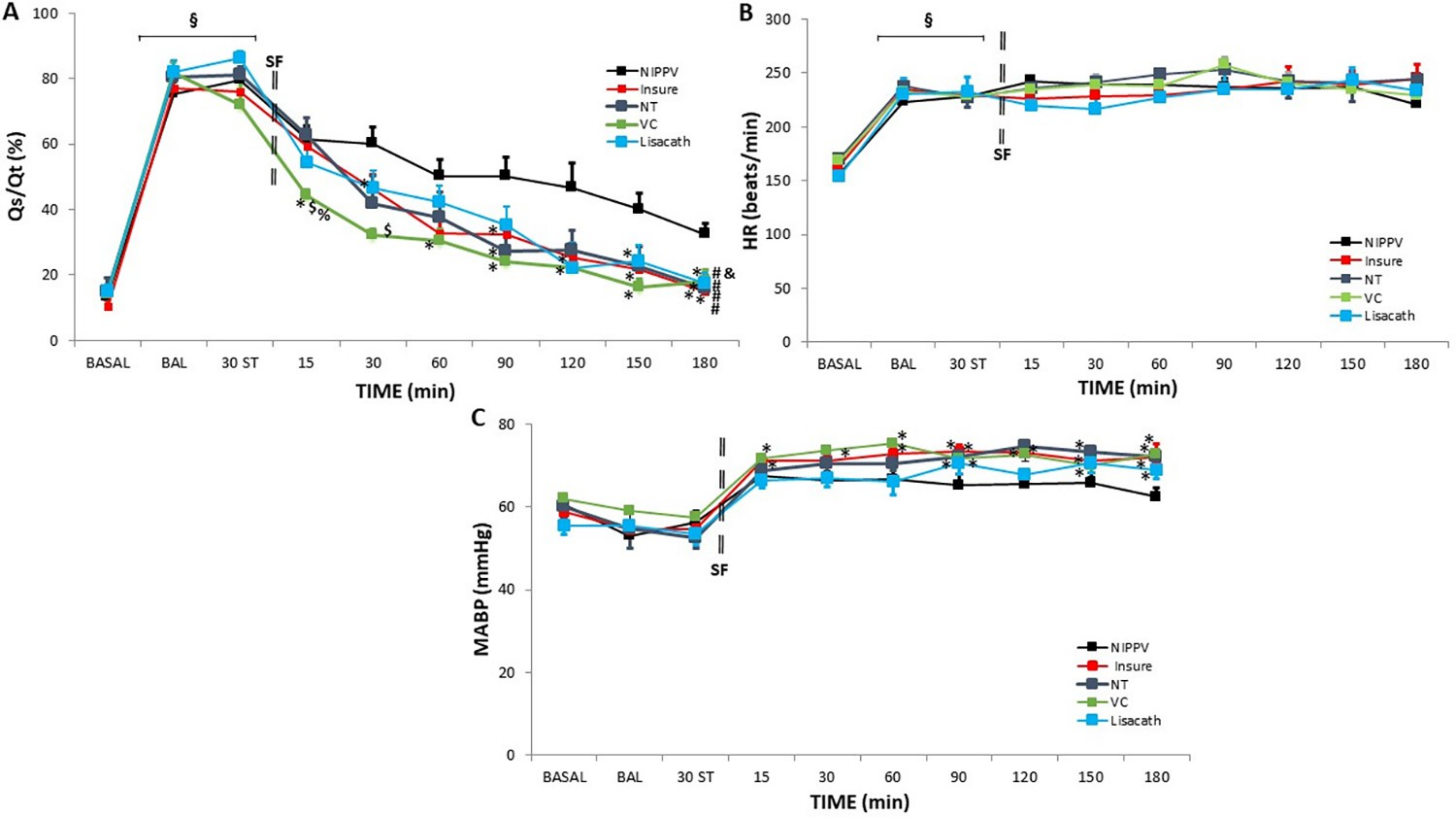
Intrapulmonary shunt ratio (Qs/Qt), heart rate (HR) and mean arterial blood pressure (MABP) in newborn piglets with bronchoalveolar lavage-induced respiratory distress treated with nasal intermittent-positive-pressure-ventilation (NIPPV) without SF therapy or with SF therapy, using the INSURE-like method, LISA with a nasogastric tube (NT), LISA with a vascular catheter (VC) or LISA with the LISAcath® catheter during the 180-min treatment period. Mean Qs/Qt (A), HR (B) and MABP (C) values in the NIPPV (black), INSURE (red), NT (dark-blue), VC (green) and LISAcath (blue) groups. (§)p<0.05 vs baseline; (*)p<0.05 vs NIPPV group, ($)p<0.05 vs INSURE group and (%)p<0.05 vs NT group (one-way-ANOVA); (#)p<0.05 vs NIPPV alone group and (&)p<0.05 vs INSURE group (two-way-ANOVA). Values = mean±SEM. ST: stabilization.

Following the BALs, significant increases were observed in Qs/Qt (Fig 4A, S6 File) and HR (Fig 4B, S7 File), while there were no significant changes in MABP (Fig 4C, S7 File) or the indicators of systemic oxygen metabolism (Table 1, S6 File). In the NIPPV alone group, though Qs/Qt gradually improved, it did not return to baseline (Fig 4A), whereas Qs/Qt did recover to baseline by the end of the experiment in all SF-treated groups (Fig 4A). Over the study period, HR (Fig 4B) and OD (Table 1) values remained similar in all groups studied and VO_2_ (Table 1) only showed short-term changes, but MABP in SF-treated groups increased to significantly higher values than in the NIPPV alone group (Fig 4C).

### Cerebral assessment

**Fig 5:**
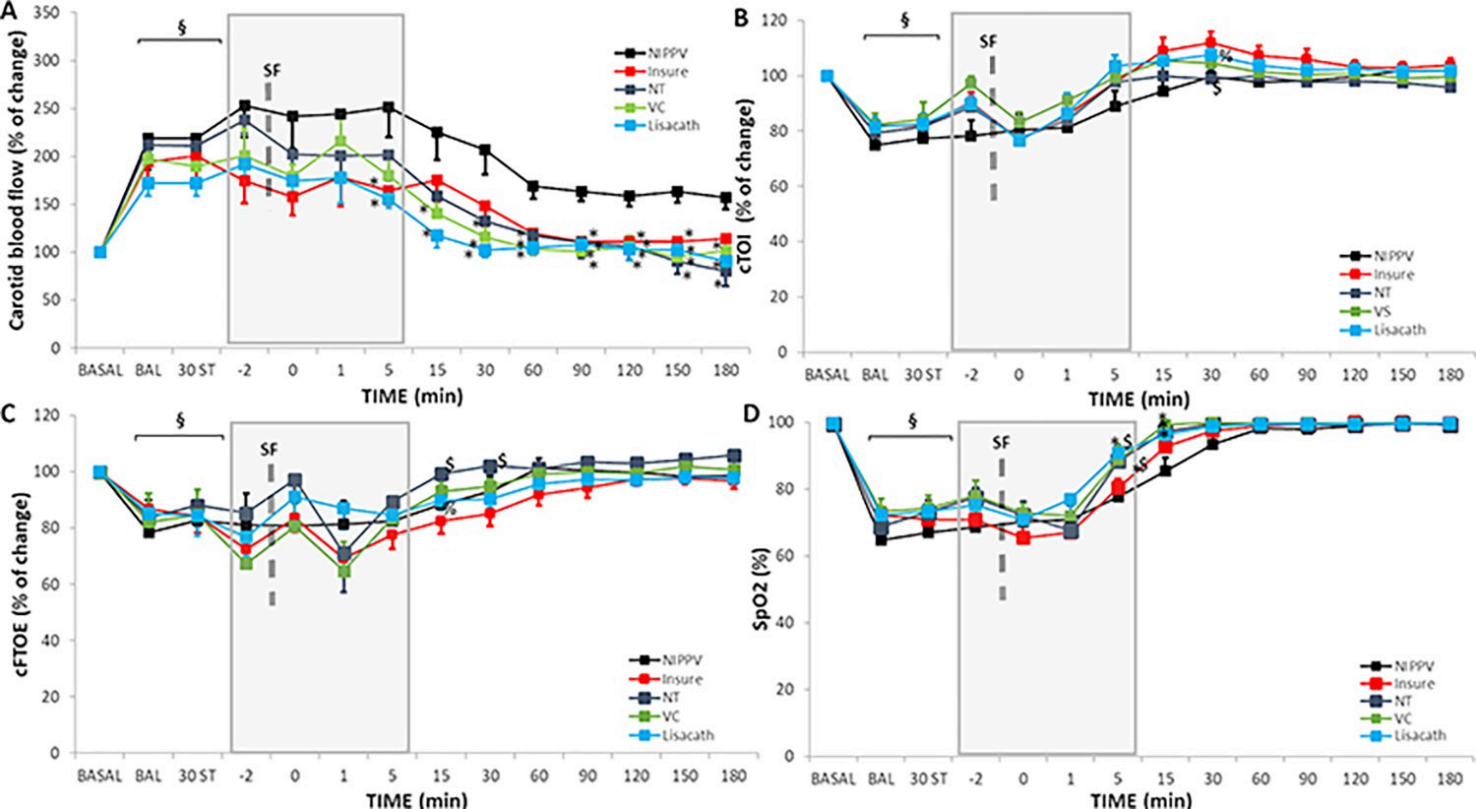
Carotid blood flow, cerebral tissue oxygenation index (cTOI), cerebral fractional tissue oxygen extraction (cFTOE) and pulse oximetry (S_pO2_) in newborn piglets with bronchoalveolar lavage-induced respiratory distress treated with nasal-intermittent-positive-pressure-ventilation (NIPPV) without SF therapy or with SF therapy, using the INSURE-like method, LISA with a nasogastric tube (NT), LISA with a vascular catheter (VC) or LISA with the LISAcath® catheter during the 180-min treatment period. Mean carotid blood flow (A), cTOI (B), cFTOE (C) and S_pO2_ (D) values in the NIPPV alone (black), INSURE (red), NT (dark-blue), VC (green) and LISAcath (blue) groups. The -2 time-point represents the 2min before SF administration and 0 time-point following completion of SF administration. (§)p<0.05 vs baseline; (*)p<0.05 vs NIPPV alone group, ($)p<0.05 vs INSURE group and (%)p<0.05 vs NT group (one-way-ANOVA). Values = mean±SEM. ST: stabilization.

Carotid blood flow increased significantly after the BAL procedure (Fig 5A, S8 File), while cTOI (Fig 5B, S8 File), cFTOE (Fig 5C, S8 File), and S_pO2_ (Fig 5D, S6 File) decreased in all groups. Subsequently, carotid blood flow decreased steadily in SF-treated groups, returning to baseline by 1 hour after the treatment, while in the NIPPV alone group, values remained higher throughout the study period.

Immediately after SF administration (t = 0), all SF-treated groups experienced a non-significant decrease in cTOI and S_pO2_, while cFTOE increased from t = -2. At 1 minute after SF administration, cTOI, cFTOE, and S_pO2_ all increased, with only short-lived differences at 5, 1,5 and 30 min after SF treatment in S_pO2_ and/or cFTOE, values being higher in LISAcath and NT groups than INSURE and/or NIPPV alone groups. Baseline was reached by 60 min after SF treatment and no significant differences were observed between groups.

Further, in general, all groups obtained low brain injury scores. Specifically, although somewhat less necrosis was observed in the LISAcath group than the other groups, scores for necrosis, edema, hemorrhage, inflammation, and infarction were low for all regions studied and did not vary significantly across the groups (Table 4 and Fig 3; S9 File).

**Table 4:** Total brain injury scores.

Groups	Necrosis	Edema	Inflammation	Hemorrhage	Infarct
**NIPPV alone**	5 (0–14)	0.13 (0–1)	0.02 (0–1)	0	0
**INSURE**	5 (0–13)	0.15 (0–1)	0.06 (0–1)	0	0
**NT**	5 (0–14)	0.13 (0–1)	0.15 (0–1)*	0	0
**VC**	5 (0–12)	0.13 (0–1)	0.22 (0–1)*	0	0
**LISAcath**	3 (0–7)*^$^%	0.19 (0–1)	0.07 (0–1)	0	0

Total brain injury scores measured in newborn piglets with bronchoalveolar lavage-induced respiratory distress treated with nasal intermittent positive pressure ventilation (NIPPV) without SF therapy or with SF therapy, using the INSURE-like method, LISA with a nasogastric tube (NT), LISA with a vascular catheter (VC) or LISA with the LISAcath® catheter after the 180-min treatment period. Values are expressed as mean±SEM. (*) p<0.05 vs NIPPV group, ($) p<0.05 vs INSURE group, (%) p<0.05 vs NT group (one-way analysis of variance). NT: nasogastric tube; VC: vascular catheter.

## Discussion

In our model of SF-deficient lung injury based on spontaneously breathing newborn piglets with BAL-induced respiratory distress, SF administration during NIPPV using different thin catheters (NT, VC, or LISAcath®) resulted in similar cerebral oxygenation to that observed when SF was administrated using the INSURE-like method. Moreover, all groups that received SF showed clinically-relevant improvements in oxygenation and other acute physiological parameters, but similar pulmonary, hemodynamic, and lung behavior. On the other hand, SF delivery using the VC and LISAcath® catheter maximized SF therapy, this being associated with better lung compliance than NIPPV alone, showing the lowest values of lung injury scores.

The administration of SF is among the most effective treatments for neonatal RDS. The INSURE method requires intubation and a period of mechanical ventilation that, though brief, may increase the risk of bronchopulmonary dysplasia [22]. Seeking to avoid the adverse effects of mechanical ventilation on premature lungs, over recent years, the use of LISA during NIV has been evaluated. Among the different NIV techniques, both animal and clinical studies have observed positive results using LISA plus NCPAP ventilation [1–4]. On the other hand, there is some evidence that the positive effects of using NIV may be enhanced if NIPPV is used instead of NCPAP [6, 14, 23, 24], and this is likely due to NIPPV being able to deliver time-cycled positive pressure ventilation above PEEP in the absence of an endotracheal tube, providing the benefits of NCPAP with less work of breathing [25]. Indeed, the benefits of NIPPV were observed in a small randomized study in preterm infants, with a reduction in the need for mechanical ventilation and SF treatment in the first 72 hours after birth [6].

Our study was designed to assess the benefits, efficacy and possible adverse effects on the brain of the administration of SF during NIPPV using different types of catheters (NT or VC, thin catheters used for LISA technique [2, 3, 11, 26], and the Ad hoc designed LISAcath® catheter), in spontaneously breathing newborn piglets with SF-deficient lung injury. Better oxygenation, intrapulmonary shunt, MABP and cerebral blood flow were observed in animals treated with LISA or INSURE-like methods, than in NIPPV controls. Similarly, in a recent randomized control trial, Gupta et al. [5] detected no differences in clinical outcomes when LISA using an NT was compared with INSURE followed by NIPPV. Although the oxygenation and hemodynamic benefits observed after SF administration seem to be similar regardless of the catheter used, only the placement of VC and LISAcath® catheters was associated with better lung mechanics than NIPPV alone. Although all groups showed normal physiological ranges of total lung injury score and biochemical lung analysis, VC and LISAcath® catheters showed the lowest values of lung damage than the other evaluated groups. The reason for this is unknown, but may be related to Magill forceps not being required when VC or LISAcath® catheters are used, it being easier and faster to place these catheters in the trachea [27], and this may decrease the SF reflux and increase the amount of SF that reaches the lung [28]. In addition, the somewhat better results with the VC and LISAcath® catheters may also be related to the fact NIPPV was used during SF administration [5, 6], as this may allow some pressure delivery down to the alveoli, which does not reach this depth if NCPAP is used during LISA [29].

No significant changes were observed in either HR or systemic oxygen metabolism in association with LISA during NIPPV, regardless of the catheter used [1], no bradycardia or apnea being observed. Subsequently, although the HR did not vary significantly in any of the groups, MABP and VO_2_ were higher in the groups administered SF than that given only NIPPV, though as in our previous studies, both values stayed within normal physiological ranges [1, 14].

In many studies, the use of a thin catheter technique for SF administration has been associated with lower rates and severity of brain injury (intraventricular hemorrhage/periventricular leukomalacia) and better neurodevelopmental outcomes in infants [3, 30, 31], probably due to few episodes of hypoxia, and less fluctuation in blood pressure and cerebral blood flow [32]. Moreover, such a technique may protect preterm infants against adverse neurodevelopmental outcomes at 18 months observed after prolonged hypoxemia [8]. In our study, in all SF-treated groups (NT, VC, LISAcath and INSURE), similar non-significant transient decreases in cerebral blood flow, S_pO2_, and cTOI, and increases in FTOE were observed immediately after SF administration during NIPPV. Moreover, by 30–60 min after SF administration, cTOI, FTOE, S_pO2_ and carotid blood flow recovered to baseline values. In addition, although some differences in brain injury scores were found between groups, this fact might not be relevant due to the follow-up of the study was relatively short (3 hours), and in the neonatal brain, acute lesions could be reversible over time.

In our previous study [1], using the same experimental model but LISA during NCPAP ventilation instead of NIPPV, short-term decreases in cerebral oxygenation were associated with SF administration by INSURE-like or LISA using an NT, while no cerebral oxygenation changes occurred with LISA using the LISAcath® catheter. Among previous clinical studies on LISA using an NT for SF administration during NCPAP ventilation, one detected transient cerebral effects, with transient impairment in cerebral autoregulation (LISA being superior to INSURE in terms of the duration of the effect) [33], while others only observed a transient decrease in rcSO_2_ during LISA and SF administration by INSURE procedures, especially in the LISA group [34], or no changes [11]. Only a crossover randomized clinical trial in premature newborns <1500 g revealed that applying NIPPV was related to lower levels of rcSO_2_ than those observed when they were under NCPAP [35], this effect not being observed in our study where NIPPV was applied as an initial treatment and then maintained throughout the experimental period. To our knowledge, this study provides the first data on cerebral oxygenation measures during NIPPV plus SF administration (using LISA with one of three different thin catheters or the INSURE-like method).

### Limitation

We recognize limitations in this study. First, our model was based on newborn piglets (2–4 days old), rather than premature piglets. For research into neonatal RDS, while models using premature animals are clinically relevant in terms of preterm neonatal physiology, it has been demonstrated that models of acute pulmonary failure can be successfully produced in both adult and juvenile animals by SF washout using repeated lung lavage [18, 36, 37]. Second, piglets have nasal, pharyngeal and cerebral vasculature anatomy that is different from that of human infants. On the other hand, the newborn piglet model does have advantages; in particular, at birth, piglets’ brain maturation, lung volume, and weight tend to resemble those of newborn infants of 36–38 weeks of gestation. Further, in this study, a temporal sham-operated control group (intubate and extubate to NIPPV without BALs) was not evaluated, our INSURE-like method is adapted to the animal protocol and may show variations compared with the clinical setting, and finally, animals were only followed for up to 3 hours, which may preclude detailed examination of histological patters and post-transcriptional and post-translational changes of inflammatory responses to the intervention. Finally, the number of animals per group may be a limitation specially for those results with small differences. Nevertheless, though animal models help bridge the gap between laboratory research and clinical practice, caution should be exercised in extrapolating results from this type of experiment to infants with RDS. Lastly, it is worth noting that the LISAcath® has been unexpectedly withdrawn from the market while writing this manuscript.

## Conclusion

This study in spontaneously breathing newborn piglets revealed that cerebral oxygenation parameters after receiving LISA (using an NT, VC or LISAcath® catheter) during NIPPV are similar to those after receiving SF by the INSURE-like method. Furthermore, taking into account the pulmonary effects, VC and LISAcath® seem to be associated with better lung outcome than the other thin catheter used for SF administration in this study. Nonetheless, longer studies and clinical trials are required to assess neurodevelopmental outcomes.

## Supporting information

S1 File VC and LISAcath. (DOCX)

S2 File Arterial and venous gas exchanges. (XLS)

S3 File Respiratory parameters and lung compliance. (XLS)

S4 File Lung biochemical analysis. (XLSX)

S5 File Lung injury score. (XLS)

S6 File Oxygen metabolism. (XLS)

S7 File Hemodynamic parameters. (XLSX)

S8 File Cerebral NIRS values. (XLS)

S9 File Brain injury score. (XLSX)
