# Supplementary material for: Structural and haemodynamic evaluation of less invasive surfactant administration during nasal intermittent positive pressure ventilation in surfactant-deficient newborn piglets
Source: PLoS One. 2023 Apr 28;18(4):e0284750. doi: 10.1371/journal.pone.0284750 (PMC10146512; doi:10.1371/journal.pone.0284750)
Supplement: S1 File — (DOCX) [file pone.0284750.s001.docx]

|  | Vascular catheter | LISAcath |
| --- | --- | --- |
| Material Composition | ------- | ------ |
| Shape of tip catheter | Rounded | Rounded |
| Surfactant flow | Easy | Easy |
| Insertion | Easy | Easy |
| Position of tip in the trachea | 1 cm beyond vocal cords | 1 cm beyond vocal cords |
| SF administration duration | One minute | One minute |
| Surfactant reflux | 1 of 6 animals | 1 of 6 animals |
